# Reasons for and Consequences of Low Energy Availability in Female and Male Athletes: Social Environment, Adaptations, and Prevention

**DOI:** 10.1186/s40798-020-00275-6

**Published:** 2020-09-10

**Authors:** Paulina Wasserfurth, Jana Palmowski, Andreas Hahn, Karsten Krüger

**Affiliations:** 1 Institute of Food Science and Human Nutrition, Leibniz University Hannover, Am Kleinen Felde 30, [PW1] 30167 Hannover, Germany; 2 Institute of Sports Science, Department of Exercise Physiology and Sports Therapy, Justus-Liebig University Giessen, Kugelberg 62, 35394 Giessen, Germany

## Abstract

Low energy availability (LEA) represents a state in which the body does not have enough energy left to support all physiological functions needed to maintain optimal health. When compared to the normal population, athletes are particularly at risk to experience LEA and the reasons for this are manifold. LEA may result from altered dietary behaviours that are caused by body dissatisfaction, the belief that a lower body weight will result in greater performance, or social pressure to look a certain way. Pressure can also be experienced from the coach, teammates, and in this day and age through social media platforms. While LEA has been extensively described in females and female athletes have started fighting against the pressure to be thin using their social media platforms, evidence shows that male athletes are at risk as well. Besides those obvious reasons for LEA, athletes engaging in sports with high energy expenditure (e.g. rowing or cycling) can unintentionally experience LEA; particularly, when the athletes’ caloric intake is not matched with exercise intensity. Whether unintentional or not, LEA may have detrimental consequences on health and performance, because both short-term and long-term LEA induces a variety of maladaptations such as endocrine alterations, suppression of the reproductive axis, mental disorders, thyroid suppression, and altered metabolic responses. Therefore, the aim of this review is to increase the understanding of LEA, including the role of an athlete’s social environment and the performance effects related to LEA.

## Key Points

- Reasons for low energy availability (LEA) are manifold and may range from unintentional undereating to severe eating disorders. The dietary behaviour of an athlete can be affected by the exercise practice environment. In addition, new challenges from the use of social media have arisen.
- Adaptations associated with LEA are known to negatively influence muscular adaptations in both endurance and strength and power athletes. Endurance athletes, because of a negative impact on mitochondrial protein synthesis and strength, and power athletes, because of a negative impact on muscle protein synthesis.
- Underperformance due to LEA may not always be noticeable as it can be masked by the positive influence of lower body weight in some sports. Athletes experiencing LEA either increase, stagnate, or decrease performance, depending on the intensity of LEA adaptation and importance of body weight on their performance. If not recognised, LEA can lead to severe health issues that can affect the ability to practice and compete.

## Introduction

It is undeniable that a lower body weight has beneficial effects on athletic performance in distance running velocity, jump height, or aesthetics. However, depending on the sport, the respective body shape and also perceived “ideal” body image varies: While athletes from endurance-based sports commonly seek low body fat and an overall thinner appearance, athletes from strength-orientated and aesthetic sports aim for low body fat and high muscle mass [1]. To achieve the desired outcome, athletes more often go on a variety of diets in an attempt to lose weight [2, 3]. Consequentially, athletes were shown to have a high relative weight variability, body dissatisfaction, and a higher frequency of eating disorders [1, 4, 5].

The pursuit of a certain body image or lower body weight to increase performance may result in low energy availability (LEA)—a state in which the body does not have enough energy to support all physiological functions needed to maintain optimal health. However, athletes may also unintentionally run into LEA during periods with increased training volume or when engaging in sports with high energy expenditure (e.g. rowing or cycling). In the context of exercise-related health risks, LEA has been extensively described in female athletes as a part of the female athlete triad in relation to bone health and menstrual function [6]. Recently, the (IOC) has expanded the triad concept with the term relative energy deficiency in sports (RED-S) to address the accompanying consequences with LEA on health and performance in both sexes [7]. Despite gaining more attention in research, many athletes and their coaches are still not aware of the health consequences of LEA related to the RED-S syndrome or are not aware of the syndrome at all [8, 9]. The need for a better understanding of LEA and RED-S is also reflected by the low priority this topic was found to have within sports federations on the international level [10]. Further, with regard to the female athlete triad, research has shown that coaches often care much more for high performance rather than preserving the long-term health of the athlete [11–13]. Beyond this background and the direct impact of the coach’s behaviour on the athletes’ health, including their dietary behaviour, coaches need more knowledge about how to act more responsibly and to think beyond “performance only”. This is evidenced by female elite runners who now fight against grievances in female sports using the hashtag #fixgirlssports. However, aside from the coach, an athlete’s health can also be largely influenced by his or her social environment (e.g. teammates) and, in this day and age, also by social media platforms.

LEA—whether unintentional or not—may have detrimental consequences on health and performance, because both short-term and long-term LEA induces a variety of maladaptations such as endocrine alterations, suppression of the reproductive axis, mental disorders, thyroid suppression, and altered metabolic responses. As the reasons for and consequences of LEA across both sexes are manifold, the aim of this review is to increase the understanding of LEA, including the role of an athlete’s social environment and the performance effects related to LEA. We discuss the LEA risk factors influenced by the athletes’ direct training environment and explain the physiologic factors and their impact on the athletes’ performance. Our suggestions could help to create a better training environment that supports long-term health and exercise participation.

## Energy Balance and Energy Availability

The foundation of an appropriate diet with sufficient intake of macro- and micronutrients that will cover an athlete’s needs is formed by adequate energy intake. Energy balance is achieved when dietary energy intake matches total energy expenditure. Furthermore, energy availability (EA) is defined as:

$$ \mathrm{EA}=\frac{\mathrm{Dietary}\ \mathrm{energy}\ \mathrm{intake}\ \left(\mathrm{kcal}\right)-\mathrm{Exercise}\ \mathrm{energy}\ \mathrm{expenditure}\ \left(\mathrm{kcal}\right)\ }{\mathrm{Fat}\ \mathrm{Free}\ \mathrm{Mass}\ \left(\mathrm{kg}\right)} $$

which equals the dietary energy left after exercise [7]. Low energy availability occurs when either dietary energy intake is too low or energy expanded through exercise is too high, leading to an insufficient amount of energy left to maintain normal physiological functions such as metabolic and immune function, bone health, and the menstrual cycle in female athletes [14, 15]. Although, to date, there are no guidelines prescribing an optimal EA for high performance athletes, studies on habitually sedentary, normal-weight women defined 45 kcal/kg fat-free mass (FFM) as a threshold at which optimal energy balance can be achieved [16, 17]. In contrast, a study on exercising men by Koehler et al. used 40 kcal/kg FFM as a threshold for a balanced EA and showed that this was still enough to support energy balance [18]. However, an EA of 30–45 kcal/kg FFM is already considered a reduced EA, and athletes should only stay within this range for a short period of time, e.g. when aiming to reduce body weight [19]. In any case, clinical studies showed that an EA of < 30 kcal/kg FFM appears to be a threshold at which severe health implications can be observed after only 5 days in healthy young women [17, 20, 21]. Therefore, low EA is commonly defined as EA < 30 kcal/kg FFM. For males, Fagerberg described a prolonged EA < 25 kcal/kg FFM as critical [22]. Nonetheless, despite those attempts to describe precise cut-offs at which symptoms low EA can be observed, individual factors influencing energy availability in an athlete should be considered [23].

## Reason for LEA and Prevalence in Different Sports across Sexes

Reasons for the development of LEA are manifold [21]. Changes in dietary intake are often a result from some form of body dissatisfaction and the desire to change the body composition. Body image satisfaction and dissatisfaction were studied in athletes compared to non-athletes and led to inconsistent findings. While some studies reported a generally greater body satisfaction in athletes compared to non-athletes, some reported the opposite, particularly when athletes compared to non-athletes came from a leanness-focused or weight class sport [24, 25]. However, it was found that elite athletes or athletes from a high-performance environment are at higher risk to experience body dissatisfaction which in turn may impact an athlete’s dieting behaviour and puts them at risk for eating disorders (ED) and consequentially LEA [1, 26, 27]. In this regard, it is important to understand that LEA can occur with or without an eating disorder [19]. Interestingly, most studies concerning body image, low energy intake, and ED primarily focused on female athletes, as it was thought that females are more susceptible to ED and LEA than males. This is also reflected by the intensively studied female triad—a condition describing the interrelation of LEA and its consequences on the menstrual cycle and bone health in female athletes [28, 29]. Although it may be true to some extent that females are more likely to experience LEA than males, growing evidence indicates that body image issues and unhealthy dietary behaviours are common in male athletes, as well [30]. In any case, restrictive dieting, ED, and the consequential LEA may not only result from body dissatisfaction but also from the belief that changes in body composition will improve performance or the social pressure to look a certain way [1].

### Dietary Behaviour and Disordered Eating

Chronic low caloric intake may result from harmless reasons, such as lack of knowledge about appropriate nutrition and the need for optimal energy balance, lack of time to prepare meals, inadequate cooking skills, and financial or even physiological reasons, i.e. loss of appetite after a training session [31]. However, the boundaries between unintentional LEA and the development of an ED are marginal and fluid, e.g. small dietary changes that were started for weight loss can become compulsive [32]. Further, additional factors, including the development of body dissatisfaction or the belief that the athlete needs to be “thin to win”, can also manifest in disordered eating [2]. Although knowledge about nutrition is more accessible than ever, and athletes were shown to generally have a better understanding of nutrition than non-athletes, many misbeliefs, such as “carbohydrates will make you gain weight” or “food intake should only occur within certain time windows”, are still common [33–35]. Overall, insufficient knowledge of general sports nutrition in athletes is still evident [31, 36, 37]. Believing and trying some of those misconceptions, e.g. avoiding specific foods, could potentially lead to a lower energy intake than is required to maintain optimal health and performance. This is also reflected by inadequate nutrient intake, particularly with regard to carbohydrates [38, 39]. Overall, the risk to experience any form of ED was increased in athletes when compared to non-athletic controls (13.5% vs. 4.6%) [5]. Particularly athletes engaging in aesthetic, leanness-focused, or weight-sensitive sports were at a higher risk to develop disordered eating patterns than athletes from sports where body weight or shape is secondary (e.g. ball sports) [5, 26, 40]. This was also shown in a study conducted by Torstveit et al., which reported a higher prevalence of EDs in female athletes from leanness-focused sports (46.7%) in comparison to athletes from non-leanness-focused sports (19.8%) [41]. In male athletes, roughly 25% of athletes from aesthetic, leanness-focused, or weight-sensitive sports showed disordered dietary patterns. This was also closely associated with greater body fat percentages and body dissatisfaction [42]. To prevent the development of disordered eating while maintaining optimal health and increasing performance of athletes, needs-based nutritional strategies for athletes in their competitive season, as well as in their off-season, are warranted. We propose to teach athletes a flexible eating behaviour. A flexible eating behaviour that acknowledges the importance of a nutrient-dense diet, while not putting labels such as “good” or “bad” on single food groups or macronutrients (e.g. low carb diet) will support psychological and physiological health in the long run [43]. Further, staff working with athletes should be educated on how to screen and detect signs of disordered eating. If an athlete shows signs of disordered eating, he or she should be guided towards seeking psychological support from a professional. Overall, ensuring psychological support for athletes can be beneficial not only for prevention and treatment of disordered eating behaviours, but also the athlete’s overall mental health [44]. Therefore, physiological counselling should be considered an inherent part of working with athletes.

In sum, it becomes obvious that food choice and dietary behaviour of an athlete are influenced by their body image and body satisfaction. However, these are also largely influenced by their social environment. Therefore, the role of coaches, teammates, and the new challenges arising from exposure and use of social media platforms need further attention.

### Coaches’ Role

Regardless of whether working with an individual athlete or in team sports, coaches play a crucial role in an athlete’s physiological and psychological health [45–47]. Although some coaches work together with different health practitioners to ensure optimal health and performance of their athlete(s), there are still coaches that do not acknowledge the importance of their athletes’ nutrition. In fact, evidence shows that not only knowledge about the existence and symptoms of RED-S and the female athlete triad, but also general sports nutrition is poor among coaches (and athletes, as described above) [11–13, 37, 48]. Such lack of knowledge directly affects an athlete’s health. Incorrect nutritional beliefs will arise by sharing general knowledge about nutrition, by exposure to unconventional methods to lose weight, or by making weight for a certain weight class [40]. Another difficulty far beyond nutrition is inappropriate comments about an athlete’s body, e.g. when he or she has gained some weight or when the coach generally thinks that the athlete would benefit from a lighter body weight [49]. The case of the elite runner Mary Cain is one well-known example that showed how such beliefs can massively influence an athlete’s physical and psychological health. In 2019, Cain broke her silence to reveal how her former coach Alberto Salazar excessively pressured her to continue to lose weight. In addition to negative comments about her appearance and body shaming in front of her teammates, Cain also reported about extreme methods to keep her thin (e.g. intake of diuretics). Consequentially, she further reported that she suffered from amenorrhea and osteoporosis—both of which are consequences of prolonged LEA [50, 51]. Kong and Harris showed that more than 60% of the elite athletes from leanness- and non-leanness-focused sports reported feeling pressure from their coaches with regard to their body [52]. Depending on the psychological health of an athlete, exposure to negative comments or drastic methods to lose weight may lay the foundation for body dissatisfaction, low energy intake, and eventually disordered eating in the long run [53]. Moreover, coaches should aim to think beyond “performance only” and keep a critical eye on an athlete’s body weight and dietary behaviour, in particular in sports where athletes think they could perform better with a lower body weight, e.g. running.

A healthy coach-athlete relationship is neither athlete- nor coach-centred but is viewed as the type of relationship that leads to mutual benefit for both parties and can ultimately also lead to optimal performance [54]. However, this should also include appropriate nutrition and nutritional strategies for the competitive- and the off-season. If needed, coaches should collaborate with dietitians to help their athletes reach their full potential without sacrificing their health.

### Teammates’ Role

Another factor that should be considered is the mutual influence among teammates—regardless of team sports or among individual athletes that are trained by the same coach. Although some relationships among teammates may be based on friendship, some may also be based on competitiveness and the urge to be “better than the other”. Especially in weight-sensitive sports, weight loss of one athlete may influence the dieting behaviour of the others. In the desire to be “better than her/him”, athletes can be influenced by the thought that “If she/he is losing more weight, then I need to lose some too!” or the perception that they are “bigger” than their teammates [55]. Furthermore, teammates can share more drastic methods to lose weight [56]. They can begin with the simple recommendation to skip meals, up to encouraging one another that it is okay to “throw up” when one has eaten too much. Reel and colleagues showed that among female college athletes, teammates had a slightly higher impact on perceived pressure concerning their weight than coaches (36.5% vs. 33.8%) [57]. Therefore, the relationship between teammates should not be overlooked when trying to identify the root cause for a lower energy intake.

### The Role of Social Media

Far beyond potential pressure from the immediate environment, athletes in this day and age are largely influenced by media and social media platforms. Particularly in regard to body image, growing evidence has shed light on the harmful effects of media exposure on both sexes [58, 59]. However, despite the promotion of “thinspiration” or “fitspiration” content, new challenges from the use of social media platforms may arise.

Athletes will compare their current size and shape to what they see on social media. Many athletes tend to post favourable pictures [60, 61]. If athletes choose to predominately post pictures in flattering poses or their “top form”, it may lead to the impression that the respective athlete can keep the same shape all year round. Unintentionally, this may be putting pressure on other athletes to keep their top form as well without acknowledging that changes in body composition between the competitive and the off-season are normal and, in some cases, (e.g. in bodybuilding) necessary. Additionally, athletes are likely to compare meals, training volume, and load to their opponents’ social media posts. In line with the findings from Vogel et al., athletes exposed to profiles of other athletes that seem to have superior positive characteristics may experience a negative influence on their self-esteem [62].

Lastly, athletes are often victims of body shaming and cyber bullying [63]. The smallest changes in an athlete’s body shape, such as weight gain, will engender many comments by the social media crowd. Well known cases of body shaming occurred after the 2016 Olympic Games, when the Mexican gymnast Alexa Moreno and the Ethiopian swimmer Robel Kiros Habt were publicly body shamed for not meeting the “lean body image” that was expected from Olympic athletes [63, 64]. Taken together, negative comments on appearance can negatively impact body image, body satisfaction, and ultimately also influence dietary behaviour, and support the development of LEA, which in severe cases can be accompanied by an eating disorder [65, 66].

On the other side of the spectrum, social media can also be used by athletes themselves to draw attention to grievances within the sporting community. After elite runner Mary Cain shared her story, other female athletes raised their voices and shared their stories. Using their social media platforms, the female runners wanted to draw attention to grievances in female sport and advocate that coaches should think far beyond “body weight only” and put an athlete’s health first [67].

## Adaptations to Short-Term and Long-Term LEA

Short-term LEA causes a disturbance of metabolic homeostasis in athletes [68]. Early human and animal studies found that fuel is spared at the cost of growth and reproduction to maintain cell survival in times of energy deprivation, [69]. Thus, metabolic mechanisms to conserve energy are evident in male and female athletes in response to prolonged LEA.

### Energy Expenditure Adaptations

To maintain basic vital functions at rest, the human body needs a certain amount of energy known as the basal metabolic rate (BMR) or the resting energy expenditure (REE). Together with the non-resting energy expenditure (NREE), REE makes up the total daily energy expenditure (TDEE). While the REE makes up the largest portion of TDEE (roughly 60–70%), the NREE makes up a much smaller portion and can be further subdivided into the non-exercise activity thermogenesis (NEAT), the thermic effect of food (TEF), and exercise activity thermogenesis (EAT) [70].

In male endurance athletes, LEA was associated with a lower REE when compared to athletes with an adequate energy supply [71]. Moreover, as energy homeostasis is also controlled by the secretion of leptin from adipose tissue via feedback to the hypothalamus (e.g. reduce REE when fat stores are low), it is unsurprising that short-term LEA, at 15 kcal/kg FFM/day, decreased basal leptin and insulin levels of exercising men concurrently with fat loss [18]. Lower leptin levels depend solely on energy availability, as shown in male rowers [72] and exercising healthy females [16], but are also a response to chronic exercise training [73]. Hilton and Loucks found lower 24-h leptin and a lower amplitude of the diurnal rhythm of leptin when EA fell below 30 kcal/kg FFM/day [16]. In addition to reducing the BMR, lower leptin levels also suppress the thyroid, the reproductive and growth hormone axes, and the inflammatory response [74, 75].

A reduction of TDEE is also mediated by thyroid suppression. Four days of LEA, at a threshold between 19 and 25 kcal/kg LBM/day, induced low triiodothyronine (T3) in exercising women who were previously inactive [76]. In contrast, males are more robust to short-term LEA as indicated by no significant alteration of free T3 concentration after five days of LEA at 15 kcal/kg FFM/day [18]. However, 3 weeks of caloric restriction attenuates T3 and NEAT in lean healthy non-exercising males [77]. In general, chronic exercise training induces a light physiologic rise of thyroid hormones in elite strength athletes and female endurance runners [78, 79], which may, to a certain degree, counteract the reduction of T3 and NEAT.

**Fig. 1:**
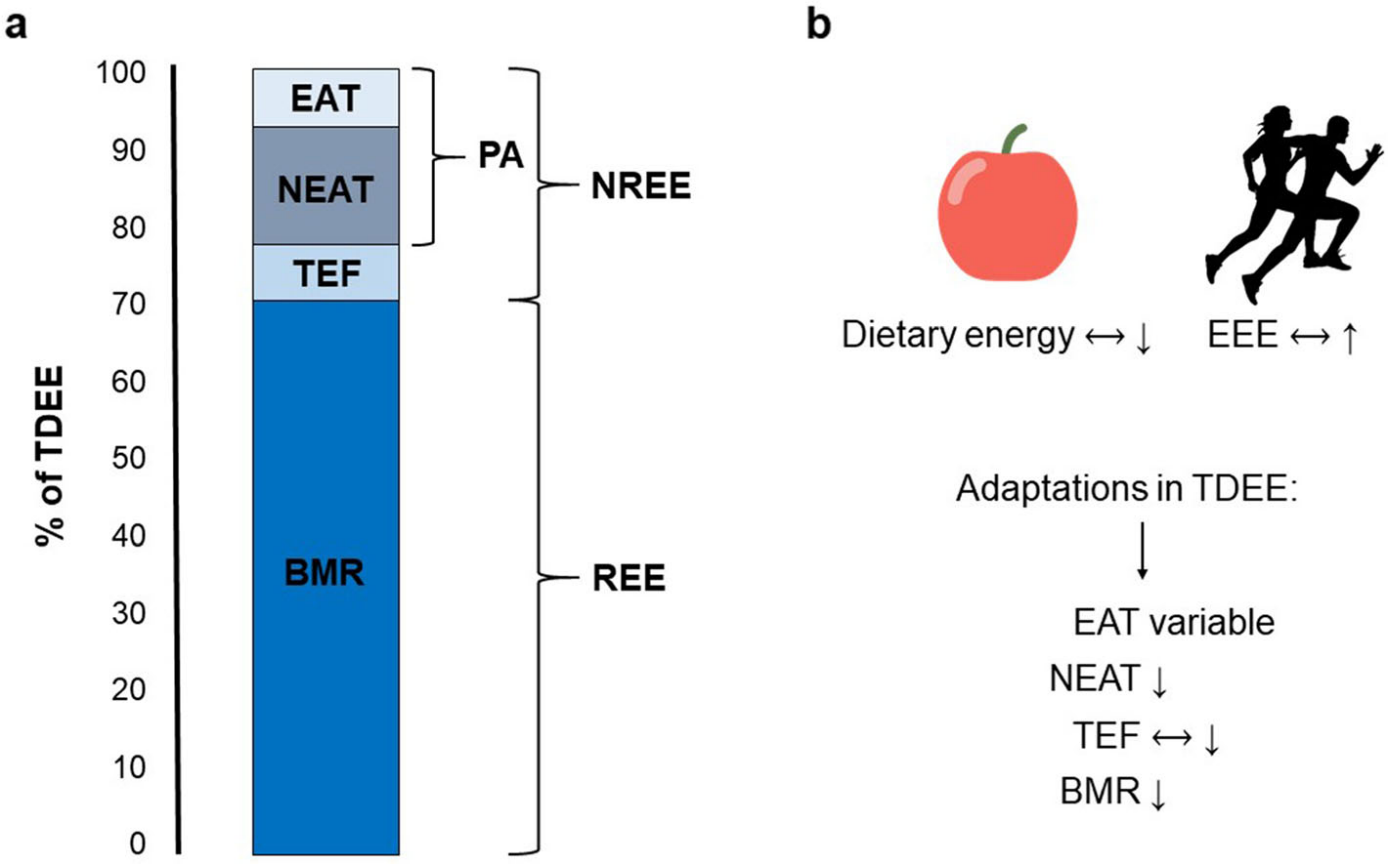
Components of total daily energy expenditure and adaptions to low energy availability are shown. **a** Total daily energy expenditure (TDEE) consists of the resting energy expenditure (REE) and the non-resting energy expenditure (NREE). NREE can be further subdivided into the thermic effect of food (TEF), non-exercise activity thermogenesis (NEAT), and exercise activity thermogenesis (EAT). Of those components, NEAT and EAT describe energy expended through physical activity (PA). **b** When energy availability is low, either by restricted dietary energy intake or increased energy exercise expenditure (EEE), metabolic adaptions to conserve energy occur. Those encompass a decline in basal metabolic rate (BMR), NEAT, and, if caloric intake is restricted, also in TEF. Generally, EAT will decrease as well but may be elevated in individuals increasing their training volume. Therefore, adaptations in this component are variable. Figure modified according to MacLean et al. [70]

Taken together data suggest, that as energy availability declines, whether intentional through caloric restriction or unintentional through increased exercise energy expenditure, metabolic adaptions will occur (Fig. 1). Although those alterations are normal and negligible if athletes return to an appropriate energy intake, e.g. after a structured dieting phase, it may be problematic in individuals that have a constant drive towards getting thinner and/or leaner. While body weight will drop at the beginning of a dieting phase, a plateau in weight loss will inevitably occur after prolonged low energy intake [68]. Although this is a normal physiological adaption, some athletes may start to further decrease energy intake to continue to lose weight. This behaviour will lead to a downward spiral of caloric restriction, losing weight, and plateauing followed by another cycle—all of which will ultimately result in LEA and likely in the development of an ED.

### Changes in Blood Substrate Levels

Metabolic changes following LEA are also evident on the blood substrate level. Following short-term LEA lasting 5 days, fasting blood glucose and insulin levels decrease, while free fatty acids and glycerol increase in male athletes [18]. In a similar fashion, insulin levels decrease, while the ketone β-hydroxybutyrate (BHB) increases in females [20]. Additionally, in female athletes positive for the female triad, hypoglycaemia and hypercholesterolemia are common [19]. In opposition to the cardio-protective function of exercise, the altered cholesterol substrate levels may be unfavourable for cardiovascular health in the long term [80]. The results indicate that decreased glycolytic activity and increased lipolytic activity during LEA in athletes of both sexes occur in order to save mainly one fuel: carbohydrates. Conceivably, this is due to limited glycogen stores [81]. Reasonably, the higher lipolytic activity in very lean athletes may be a challenge for the physiologic system since high-performance athletes’ fat stores were often reported to be close to the lower limit of 5% for men and 12% for women, particularly in athletes engaging in endurance or aesthetic sports [22, 82, 83]. In one study, only 3 days of LEA reduced muscular glycogen stores and conserved energy in the form of adipose tissue in male endurance athletes [84]. Moreover, in female athletes with severe eating disorders, such as anorexia nervosa, the restriction of food and fluid intake can lead to imbalanced electrolytes, anaemia, and hypotension [19, 85]. As the impact of LEA slightly differs across sex, we further discuss reproductive and bone adaptations in separate sections.

### Sex-Specific Endocrine and Bone Adaptations

Suppression of the female athlete’s reproductive system by LEA has been described extensively within the triad research [86]. Reproductive health in females is sensitive to short-term LEA, in terms of disrupting luteinising hormone pulsatility during waking and sleeping hours when EA falls below 30 kcal/kg FFM/day [20]. LEA below 30 kcal/kg/FFM indicates a clinical menstrual status and clearly differentiates amenorrhea, defined as no menses for 90 days, from eumenorrhea in exercising female athletes [87]. Female runners with functional hypothalamic amenorrhea (FHA) express lower oestrogen levels [88]. Accordingly, rapid bone loss due to low oestrogen is associated with menstrual disorders [29]. For instance, the bone fracture risk of amenorrhoeic female elite runners was nine times higher than their healthy counterparts [89].

Furthermore, during LEA, oestradiol and progesterone are reduced in female athletes with RED-S [90]. In females, oestradiol levels are extremely sensitive to and are attenuated by within-day LEA [91]. Oestradiol preserves bone mass density (BMD) by increasing osteoclasts and decreasing osteoblast apoptosis [92]. Adequate oral administration of oestrogens can prevent a reduction in bone mass after menopause [92]. Before menopause, a lack of successful oral oestrogen therapy is likely due to the downregulation of insulin-like growth factor-1 (IGF-1) [93]. Therefore, in the young female athletes, improving LEA may have a stronger effect than oestrogen therapy. In contrast to oral oestrogens, transdermal oestrogen treatment has been effective in increasing bone mass density in amenorrhoeic female athletes [94].

The influence of LEA on BMD, apart from reproductive hormones, is highlighted by analysis of bone turnover markers [95]. Ihle and Loucks found changes in three bone turnover markers in response to short-term LEA in exercising females, namely a reduction of the bone formation markers plasma osteocalcin and serum type I procollagen carboxy-terminal propeptide, and an increase in the bone resorption marker urinary N-terminal telopeptide [96]. Extreme LEA (10 kcal/kg FFM/day) increased bone resorption markers, while formation markers declined at minor levels of energy restriction between 20-30 kcal [96]. In contrast, a more recent study found that 3 days of LEA at 15 kcal/kg LBM did not significantly alter the common bone resorption markers, such as β-carboxyl-terminal cross-linked telopeptide of type I collagen amino-terminal propeptide of type 1 procollagen, though it also lowered the bone formation marker carboxyl-terminal propeptide of procollagen type 1 [97]. The decrease in the same bone formation marker during 5 days of LEA at 15 kcal/kg LBM was verified in another study by the same group. In the same study, no significant changes in these newer bone turnover markers were found in males [95].

Exercise hypogonadal male condition (EHMC) is the syndrome affecting reproductive function of males, akin to the triad in females. During EHMC, the hypothalamic-pituitary-gonadal axis is disturbed along with reduced serum testosterone levels (TES) as a response to LEA [98]. Although TES values remain in the low end of the normal clinical range [89], symptoms of hypogonadism—fatigue, sexual dysfunction, and low bone mineral density—are present. The syndrome was first diagnosed in endurance-trained males; however, it is also seen in power athletes [98] and energy-restricted bodybuilders [99]. Of note, protein intake does not mediate TES reduction in exercising males [100]. However, current information on LEA and endocrine changes in males is based on case reports and studies with small sample sizes [101]; therefore, further research is needed.

Similar to female athletes, progesterone and oestradiol levels are reduced in response to LEA in male athletes [90]. Consistently, architecture and turnover markers of bone were significantly reduced in endurance runners [89]. For male athletes other than runners, evidence on bone turnover markers is less clear. However, there is also some evidence of lower bone health in male athletes with LEA who participate in race horse riding or cycling events [102]. In addition, young males with a low BMI sharing the belief that leanness improves performance are more likely to have low BMD [103].

Overall, the altered endocrine profile caused by LEA, including decreased anabolic hormones (e.g. leptin, oestradiol, TES) in both male and female endurance athletes, is harmful to BMD [89]. The sensitivity of bone turnover makers seems to be sex-specific and higher in female than in males when experiencing short-term LEA. Of course, there are other factors affecting BMD. The interested reader may refer to a more comprehensive review on the athletes’ bone health (see Sale and Elliot-Sale, 2019 [90]).

### Suppressed Growth Hormone Axis

**Fig. 2:**
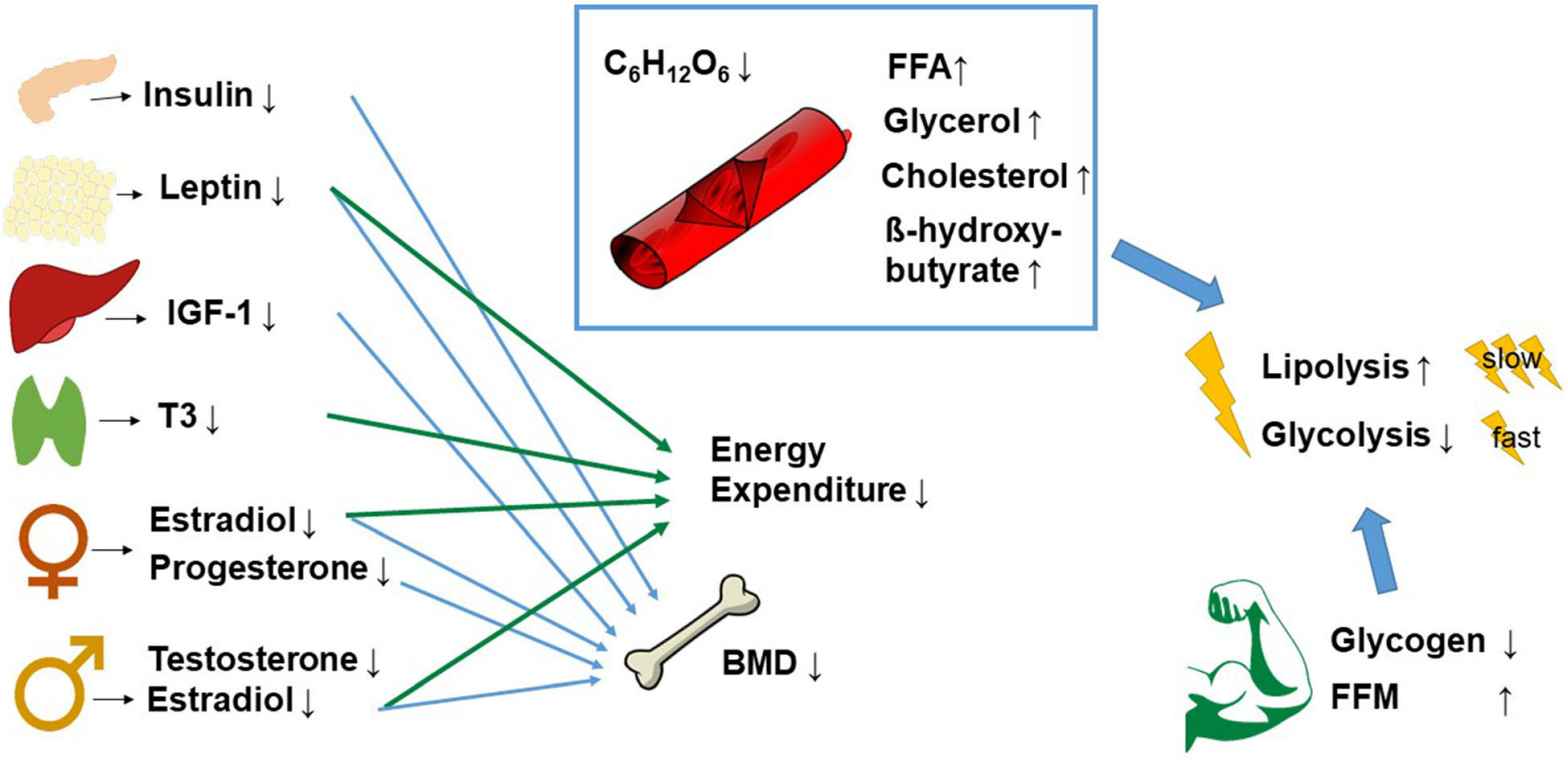
Overview of selected body-wide effects due to low energy availability (LEA). On the left, body-wide effects of LEA: lower insulin, leptin, insulin-like growth factor 1 (IGF-1), and triiodothyronine (T3) as well as lower oestradiol and progesterone in female and lower testosterone and oestradiol in male athletes. Their influence on lower energy expenditure and/or decreased bone mass density (BMD) is depicted by the arrows. In the middle, alterations in substrate: lower glucose, higher free fatty acids (FFA), higher glycerol, higher cholesterol, and β-hydroxybutyrate. These alterations, combined with lower glycogen stores and an increased percentage of fat-free mass (FFM), potentially increase lipolysis and decrease glycolysis

Other key metabolic hormones have been discussed to mediate TDEE adaptations by lowering REE such as those decreasing anabolic pathways [68]. For example, insulin-like growth factor 1 (IGF-1) mediating muscular and bone growth [104] is commonly higher in well-trained, lean subjects [105]. On the contrary, it is significantly lower when EA is reduced to 10–20 kcal/kg FFM/day in exercising women who were previously sedentary [20]. In line with the lower IGF-1, its carrier insulin-like growth factor-binding protein 3 is also decreased in females when they have LEA [20]. Overall, the concentration of IGF-1 is linked to BMD in pre- and post-menopausal women [92]. In contrast, males are more robust to short-term LEA, as IGF-1 was not significantly altered after five days of LEA at 15 kcal/kg FFM/day. However, a trend for a reduction was present [18]. Long-term caloric restriction in male lean body builders lasting 11 weeks reduced IGF-1 in the period prior to competition [99]. In contrast, growth hormone (GH) levels mediating IGF-1 are greater in male and female athletes positive for RED-S [90] and may indicate the development of a GH resistance [101]. For instance, in male power athletes with low body fat, growth hormone treatment administered in a double-blind controlled trial neither influenced body composition nor muscle strength [106]. Of note, protein intake does not mediate IGF-1 levels in exercising athletes—independent of their sex [100]. An overview of body-wide effects of LEA that lead to changes within the athlete’s body and also have an impact on performance is provided in Fig. 2.

### Immune Homeostasis

An association between LEA, impaired immunity, and infection is likely, as nutrients are also important for the immunometabolism of leukocytes [107]. However, there is currently no evidence supporting this hypothesis [108]. Athletes face multiple challenges other than LEA that suppress immune function, ranging from psychological stress to sleep deprivation [109]. Supporting of the RED-S definition, which includes impairments of immunity, is the association between LEA and upper respiratory tract infection (URTI) risk in female athletes [110]. In addition, Sarin et al. described immunosuppression after energy restriction in immune cells such as T cells and B cells [111]. Furthermore, interleukin-6 (IL-6) expression mediating inflammation is reduced by sufficient energy intake before exercise in order to spare muscular glycogen [112]. Pasiakos et al. found that increased IL-6 levels after endurance exercise are negatively correlated with energy balance and glycogen stores [113]. This change was also accompanied by increased hepcidin levels regulating iron metabolism. In this context, Badenhorst et al. assume that an increase in baseline levels of hepcidin arises when either LEA occurs for several days during high energy expenditure or when inflammation as indicated by increased baseline IL-6 levels is present [114]. Similarly, haematological constraints, such as abnormal bruising, anaemia, low haemoglobin, iron, or ferritin, are 1.6 times more likely in female athletes at risk for LEA [115]. A dysfunctional haematopoiesis may be present in female athletes with LEA, since prolonged energy restriction and intense exercise are associated with lower erythrocyte and platelet counts, while the number of white blood cells increases [111].

### Mental Aspects and Predisposition

With regard to psychological stress, anxiety and depression have a significant effect on immunity, and an attenuated resistance to infections is well described [108]. Accordingly, male endurance athletes with symptoms of LEA more often achieve high exercise dependence scores known to correlate with ED [116]. An increased drive for thinness in female endurance runners with LEA and FHA compared to athletes with normal menses has also been found [19]. Apparently, increased psychological stress on the athlete, if present, will lead to an attenuated resistance to infections. Noticeably, a mental predisposition for an increased risk of LEA or for mental consequences of LEA has to be investigated in the future.

Chronic stress increases cortisol levels, which may increase the risk of anxiety and depression [117]. Elevated cortisol levels were also found in females with triad risk factors [19], as these are closely related to augmented psychological stress during training and fasting. The cortisol change does not happen unequivocally due to severe energy restriction [118]. Furthermore, cortisol levels are highly variable throughout the day due to circadian rhythm [119]. Moreover, results on cortisol levels as evidence of associations between LEA and cortisol levels are inconsistent [90]. Again, cortisol levels in females may be more sensitive to LEA, since after mere hours of within-day LEA, levels significantly increased [91]. We need highly standardised research to clarify if changes in cortisol are a primary or secondary consequence of prolonged LEA in the future.

## Consequences for Performance

As RED-S in athletes was defined recently, there is little research on performance in regard to this specific syndrome. One study by Ackerman et al. investigated the body-wide influence of LEA in female athletes through several questionnaires [115]. Performance effects of LEA were “decreased training response, impaired judgement, decreased coordination, decreased concentration, irritability, depression, and decreased endurance performance” [115], and lower bone health. The authors did not find any evidence of immunologically harmful adaptations due to LEA. As no evidence of an attenuated immune response exists (see the “Mental aspects and predisposition” section), it is hypothesised that the immune system may be the last system to shut down. However, performance decrements are not exclusive to female athletes. In male cyclists, prolonged EA, despite higher training loads, resulted in underperformance, while there was no association between body fat and performance for this sport [120].

**Fig. 3:**
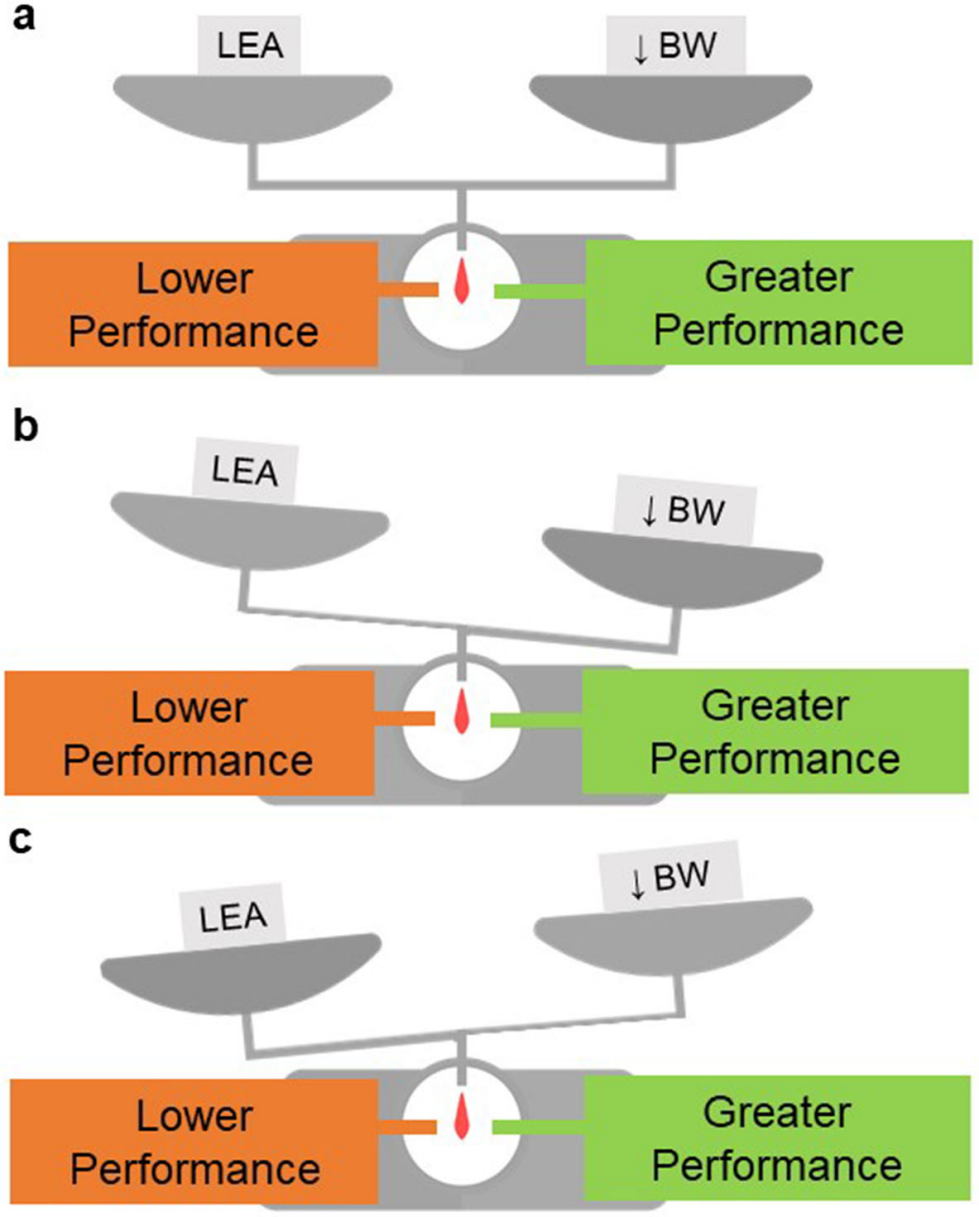
Interrelation between an athlete’s body weight, LEA and performance. **a** Depending on the influence of body weight (BW) on performance, weight loss may mask underperformance in athletes experiencing low energy availability (LEA). Despite the negative consequences of LEA on performance, athletes may stay at the same level, when the positive influence of lower body weight is equal to the negative effect of LEA on performance. **b** When the positive influence of BW on performance outweighs the negative influence of LEA on performance, athletes may even get better, although they cannot exploit their full potential. **c** However, when negative adaptations due to LEA are greater than the positive influence of weight loss, performance deficits may be clearly associated with LEA

Even though there is a dearth of direct research on performance effects of RED-S, optimal energy supply is essential to optimise athletic performance [121]. We have outlined energy-conserving mechanisms by endocrine hormones and reduced glycogen stores as some of the homeostatic adaptations to LEA in the previous section. It is hypothesised that a deficient energy homeostasis is the main cause underlying the development of overtraining [121]. Skeletal muscle, which controls locomotion, is a key regulator of metabolic homeostasis. Repetitive exercise bouts increase metabolic enzymes and protein content in the long term, whereby variations exist, depending on the placement of the exercise on the continuum between endurance and resistance exercise. Endurance exercise has a pronounced effect on mitochondrial protein content and resistance exercise has a pronounced effect on myofibrillar protein content to enable performance enhancement [122]. T3 mediates the elevated mitochondrial content in endurance athletes as it stimulates ATPase activity and increases heat production [123]. Therefore, low T3 associated with LEA reduces ATPase activity, leading to reduced energy production by mitochondria and has a negative effect on aerobic energy production and vice versa. This is why increasing NEAT and reducing body weight by T3 supplementation is a promising strategy to enhance performance [124]. On the other edge of the exercise continuum is the pronounced effect on muscle protein content. On the one hand, stimulation of muscular protein synthesis is promoted by anabolic hormones, such as insulin, IGF-1, and TES. On the other hand, catabolic glucocorticoids, such as cortisol, increase protein turnover and initiate skeletal muscle protein breakdown [125]. A negative effect on muscular protein synthesis due to LEA is implied by reduced anabolic hormones and a potential increase of cortisol in more severe or prolonged LEA. Thus, it is unsurprising that female runners with secondary FHA demonstrated a lower neuromuscular performance reflected by longer manual reaction time and significant lower knee muscular strength and endurance compared to eumenorrheic athletes [88]. In judo athletes, caloric restriction was associated with poor performance while increasing fatigue and tension and decreasing vigour, as well [126]. In addition, a decreased performance in other power athletes seems likely due to decreases in glycogen stores [81]. To summarise, muscular adaptations important to both endurance and resistance athletes are disturbed by LEA alterations. The influence of LEA on performance may be masked by the tremendous effect of body weight on performance or may even result in slight performance enhancement or stagnation (Fig. 3). A clear decrease in muscular performance may not be obvious despite the athlete underperforming.

Next to optimal energy supply, continuous training is key for high-performance athletes to maintain high training volumes in order to reach the limits of physical performance. Of course, the more days athletes report as sick days, the more training hours are reduced [127]. There is some evidence that training absence due to illness is three times higher in athletes with a risk of LEA. These athletes miss more than 22 days of training within a year, which is 3 times more than athletes at no risk [128]. However, as already mentioned, illness data is scarce. As a reduced EA negatively influenced bone health in physically active individuals [129], more evidence is found on training absence due to bone injuries of male athletes with low TES and amenorrhoeic females in runners. Injury risk was 4.5 times higher as compared to healthy counterparts [89]. With increasing risk for the female triad, the risk for bone stress injuries in a large cohort of 259 females increased significantly [130]. However, while LEA in female athletes has a negative effect on BMD, there is a concurrent performance-enhancing outcome of endocrine alterations concerning oestrogen. The associated low oestrogen increases the stiffness of connective tissue such as ligaments and tendons [128]. A higher stiffness of the connective tissue is associated with performance parameters such as jump height [131]. Therefore, the risk of ligament injuries and the power performance of women with low oestrogen potentially increases [132]. As we pointed out in the Adaptations to Short-Term and Long-Term LEA section, for male athletes other than runners, evidence on BMD is less clear. Overall, the risk of training absence may increase when exercising with LEA and thus leads to underperformance—noticeable or not.

## Conclusion

Taken together, the evidence shows that LEA causes body-wide effects paving the way for the recognition of RED-S as a multifactorial condition in athletes. Causes for LEA range from harmless reasons, such as lack of motivation to prepare meals, up to deliberate chronic undereating with severe Eds. The consequences on health and performance outlined by the female triad and the hypogonadal male condition are self-evident, though better-controlled, highly standardised trials are needed.

Given the information above, this article should highly encourage coaches to support a healthy environment during daily practice. Staff involved in the supervision of athletes should be sensitised for signs of LEA and openly talk to athletes. With regard to performance, professionals should keep in mind that performance reductions due to LEA might not necessarily come with LEA. In weight-sensitive sports, athletes may even enhance performance being at LEA due to lower body weight and a higher tissue stiffness. Beyond that, the sociocultural pressure and influence of media on athletes should not be overlooked. Yet again, more studies investigating their impact on athletes in this day and age are necessary. As evidence shows the high prevalence of feeling pressure from coaches or teammates, particularly coaches should acknowledge their impact on athletes and act responsibly.

## Data Availability

Not applicable

## Abbreviations

BMD: Bone mass density
BMR: Basal metabolic rate
EA: Energy availability
EAT: Exercise activity thermogenesis
ED: Eating disorders
EHMC: Exercise hypogonadal male condition
FFM: Fat-free mass
FHA: Functional hypothalamic amenorrhea
GH: Growth hormone
IGF-1: Insulin-like growth factor 1
IL-6: Interleukin-6
IOC: International Olympic Committee
LEA: Low energy availability
NEAT: Non-exercise activity thermogenesis
NREE: Non-resting energy expenditure
RED-S: Relative Energy Deficiency Syndrome
REE: Resting energy expenditure
T3: Triiodothyronine
TDEE: Total daily energy expenditure
TEF: Thermic effect of food
TES: Testosterone
URTI: Upper respiratory tract infection
